# Vagus Nerve Stimulation in Early Stage of Acute Myocardial Infarction Prevent Ventricular Arrhythmias and Cardiac Remodeling

**DOI:** 10.3389/fcvm.2021.648910

**Published:** 2021-04-26

**Authors:** Shuang Zhao, Yan Dai, Xiaohui Ning, Min Tang, Yunzi Zhao, Zeyi Li, Shu Zhang

**Affiliations:** State Key Laboratory of Cardiovascular Disease, Arrhythmia Center, National Center for Cardiovascular Diseases, Fuwai Hospital, Chinese Academy of Medical Sciences and Peking Union Medical College, Beijing, China

**Keywords:** vagus nerve stimulation, myocardial infarction, ventricular arrhythmia, gene expression, cardiac neural remodeling, heart failure

## Abstract

**Aims:** To evaluate whether low level left vagus nerve stimulation (LLVNS) in early stage of myocardial infarction (MI) could effectively prevent ventricular arrhythmias (VAs) and protect cardiac function, and explore the underlying mechanisms.

**Methods and Results:** After undergoing implantable cardioverter defibrillators (ICD) and left cervical vagal stimulators implantation and MI creation, 16 dogs were randomly divided into three groups: the MI (*n* = 6), MI+LLVNS (*n* = 5), and sham operation (*n* = 5) groups. LLVNS was performed for 3 weeks. VAs, the left ventricular function, the density of the nerve fibers in the infarction area and gene expression profiles were analyzed. Compared with the MI group, dogs in the MI+LLVNS group had a lower VAs incidence (*p* < 0.05) and better left ventricular function. LLVNS significantly inhibited excessive sympathetic nerve sprouting with the evidences of decreased density of TH, GAP43 and NF positive nerves (*p* < 0.05). The gene expression profiling found a total of 206 genes differentially expressed between MI+LLVNS and MI dogs, mainly involved in cardiac tissue remodeling, cardiac neural remodeling, immune response and apoptosis. These genes, including 55 up-regulated genes and 151 down-regulated genes, showed more protective expressions under LLVNS.

**Conclusions:** This study suggests that LLVNS was delivered without altering heart rate, contributing to reduced incidences of VAs and improved left ventricular function. The potential mechanisms included suppressing cardiac neuronal sprouting, inhibiting excessive sympathetic nerve sprouting and subduing pro-inflammatory responses by regulating gene expressions from a canine experimental study.

## Introduction

Acute myocardial infarction (MI) is a major cause of morbidity and mortality worldwide and continues to pose significant therapeutics challenges (1). Although timely myocardial reperfusion is the most effective therapeutic to reverse myocardial damage, the abrupt restoration of blood flow to ischemic tissue can induce ventricular arrhythmias (VAs) (2). The incidence of VAs is high especially during the first month after MI (3). Implantable cardioverter defibrillators (ICDs) have been proven to be an effective treatment to terminate VAs. However, no trials have shown benefits on long-term mortality due to ICD implantation after MI (4).

Left vagus nerve stimulation (VNS) is a safe and effective treatment for neurological disorders (5). In recent years, VNS treatment before or during ischemia has been shown an antiarrhythmic effect and protect against cardiac remodeling in animal models (6–9). However, two large clinical trials failed to demonstrate that VNS could improve cardiac function or reduce mortality (10, 11). Another study showed VNS was a promising therapy in heart failure (HF) (12–16). The interfaces and stimulation protocols used among the abovementioned studies were various, probably contributing to different results (17–19). Ardell et al. (18) described an approach to optimal dosing, yielding physiological augment of vagus nerve activity. In addition, all the published trails focused on refractory HF rather than the onset of HF.

In the present study, low level left VNS (LLVNS) using optimal dosing was performed at the onset of HF. The objective was to:1) evaluate whether LLVNS could be an effective therapeutics for preventing VAs and improving cardiac function; and 2) explore the underlying mechanisms using a canine model.

## Materials and Methods

**Figure 1:**
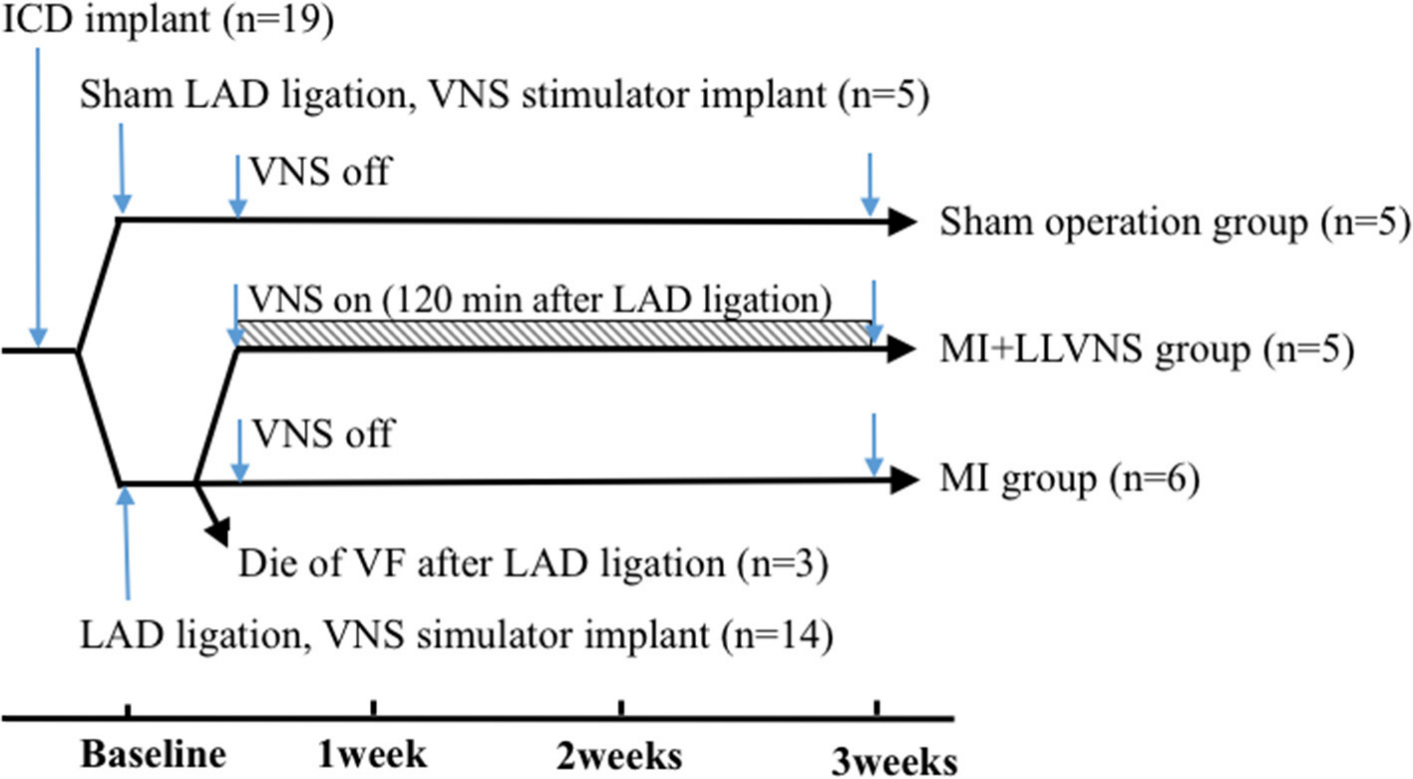
Timeline of the experimental protocol. ICD, implantable cardioverter defibrillators; LAD, left anterior descending coronary artery; VNS, vagus nerve stimulation; VF, ventricular fibrillation; LLVNS, low level left vagus nerve stimulation; MI, myocardial infarction.

The experimental preparations and protocols were approval by the Animal Care and Use Committee of the Chinese Academy of Medical Sciences, Peking Union Medical College, Fuwai Hospital and Cardiovascular Institute, Beijing, China. Adult mongrel dogs of either sex (*n* = 19, 17–22 kg) were used in this study. The investigation conforms to the Guide for the Care and Use of Laboratory Animals published by the US National Institutes of Health (NIH Publication No. 85–23, revised 1985). At the end of the experiment, all animals were euthanized with a lethal dose of sodium pentobarbital. The experimental protocol is shown in Figure 1.

### Animal Preparation

All dogs were anesthetized with 40 mg/kg sodium pentobarbital and ventilated with room air by a positive pressure respirator (ACM619, Beijing Aerospace Changfeng Co., Ltd, Beijing, China). Additional maintenance doses of 2 mg/kg sodium pentobarbital were administered at the end of each hour during the procedure. Ringer's lactate solution was continuously infused through the ear vein. Continuous body surface electrocardiograms (ECGs) were recorded. A heating pad was used to maintain the core body temperature at 36.5 ± 1.5°C. In addition, percutaneous arterial oxygen saturation (SpO_2_) and blood pressure (BP) were monitored throughout the procedures.

### Implantable Cardioverter Defibrillator Implantation

Under fluoroscopic guidance, passive fixation pacing (4574, Medtronic, USA) and defibrillation (6935, Medtronic, USA) leads were placed through the right internal jugular vein into the right atrial appendage and right ventricular apex, respectively. The leads were connected to an ICD (Protecta DR D364DRM, Medtronic, USA), and the system was subsequently implanted in the neck region. The device was maintained in the DDD mode with a lower pacing rate of 60 bpm. The arrhythmia detection zones were set at 230 bpm for ventricular tachycardia (VT) and 250 bpm for ventricular fibrillation (VF). The device therapies were enabled to deliver full device output (35 J) for VT/VF detection during the infarction and first 24 h after MI and were disabled at all other times. Counts of the VF, VT, nonsustained ventricular tachycardia (NSVT) and premature ventricular contraction (PVC) recorded by the ICD were determined for the data analysis.

### Creation of MI and Experimental Groups

A left thoracotomy was performed in the third to fourth intercostal space. The left anterior descending coronary artery (LAD) was isolated in all dogs, except for those in the sham operation group, and the occlusion was performed between the first and second diagonal branches to produce the MI model. All major diagonal branches were ligated to decrease collateral flow to the infarct area. The LAD was partially occluded for 10 min and then tied off completely. Animals in the sham operation group (*n* = 5) were served as controls, the chest cavities of them were opened without LAD isolated. MI was confirmed by the appearance of an acute ST-segment elevation in the ECG. The dogs were subsequently monitored for 60 min to detect arrhythmias. Then, the chest was closed in layers, and the pneumothorax was reduced. Three dogs died of VF that occurred immediately after the induction of LAD occlusion that was not terminated by electrical defibrillation attempts. The remaining 11 dogs were randomly divided into 2 groups [the MI (*n* = 6) and MI+LLVNS (*n* = 5) groups] with a follow-up period up to 3 weeks.

### Low Level Left Vagus Nerve Stimulation (LLVNS)

The vagus nerve in the left neck of the 16 dogs was separated, and a cervical VNS cuff electrode was inserted. The other end of the electrode was connected to a nerve stimulator (Ensense Biomedical Technology Co., Ltd., China) buried in a pocket at the neck area. In the VNS+MI group, the stimulators were turned on for 120 min after LAD occlusion with the following stimulus parameters, which were described as optimal dosing by a previous study (18): the output amplitude was set at the stimulation threshold of minus 1 volt and did not cause a heart rate (HR) change (the threshold was the output which could cause an abrupt decrease in the baseline HR by 20%); the pulse width was 0.5 ms; and the frequency was programmed at 10 Hz with continuous recurring cycles of 12 s on and 60 s off. The average voltage used for VNS was 1.2 ± 0.4 V (range from 0.8 to 1.6 V). The devices implanted in the MI and sham operation groups remained off. The HRs were recorded at baseline, before the VNS device implantation and at 1 h after the VNS device implantation.

### Echocardiographic Evaluation

Transthoracic echocardiography was performed at baseline and 3 weeks after MI. Standard 2-dimensional short- and long-parasternal views and 4-, 2-, and 3-chamber apical views were obtained via standard procedures using a 3S transducer coupled to a Vivid 7 echocardiographic machine (GE Medical, Milwaukee, Wisconsin). The left ventricular (LV) end-diastolic dimension (LVEDD), LV end-systolic dimension, and LV ejection fraction (LVEF) were measured twice according to the modified American Society of Echocardiography guidelines by two different physicians trained in ultrasound technology. The averages were used in the final data processing.

### Immunohistochemistry

Hearts were cut and fixed in 4% formalin for 48–72 h at 4°C. Sectioning was performed longitudinally (along the direction of blood flow). The segments of the peripheries of the myocardial scars and perivascular regions were processed into paraffin blocks, and oriented to clearly visualize the epicardial and endocardial surfaces. The paraffinized tissue blocks were cut into 3 μm-thick sections. For each paraffin block, one slide each was stained with hematoxylin-eosin (HE) to accentuate muscle and connective tissues. Then, immunohistochemical staining was performed to detect growth-associated protein 43 (GAP43), tyrosine hydroxylase (TH) and neurofilament (NF) (Proteintech Group, Rosemont, USA) and evaluate the nerve density according to standard procedures.

The density of the TH, NF, and GAP43 positive nerve fibers was assessed using the images of 20X magnification areas, which was obtained under a light microscope equipped with a computerized image system (Leica QWin V3, Germany). The density was expressed as the average of ten randomly selected fields (μm^2^/mm^2^). The average density of all the nerves in the field of view was regarded as the average density of nerves of the slices. Computer aided Bioanalysis Image Software 2012 (CEWEI Co., Ltd. Shanghai, China) was applied to calculate the density of the nerves.

### Gene Expression Profiling

#### Preparation of Myocardial Tissue

In accordance with previous studies, (20) we used sample pooling strategies for microarray analysis. Three dogs from the MI group and three dogs from the MI+LLVNS group were randomly chosen and sacrificed 3weeks after MI following the echocardiographic assessment. Remote zone tissues of the LV free wall were collected and stored in liquid nitrogen; then, total RNA was extracted and used for the microarray and real-time PCR experiments.

#### Microarray Data Analysis

Total RNA was isolated using a Takara RNAiso Plus Kit (Takara, DaLian, LiaoNing, CN) according to the manufacturer's instructions. RNA integrity was analyzed by standard agarose gel electrophoresis and ethidium bromide staining. Qualified total RNA was further purified by using an RNeasy Micro Kit (Qiagen, GmBH, Germany). RNA samples from each group were then used to generate biotinylated cRNA targets for the Affymetrix Canine Genome 2.0 array (Affymetrix, Santa Clara, CA, US). The array was hybridized and washed using GeneChip® Hybridization, Wash and Stain Kit (Affymetrix, Santa Clara, CA, US), a Hybridization Oven 645 (Affymetrix, Santa Clara, CA, US) and a Fluidics Station 450 (Affymetrix, Santa Clara, CA, US) according to manufacturer's instructions. Slides were scanned by a GeneChip® Scanner 3000 (Affymetrix, Santa Clara, CA, US) and Command Console Software 4.0 (Affymetrix, Santa Clara, CA, US) using default settings. Raw data was normalized by the MAS 5.0 algorithm, Gene Spring Software 12.6.1 (Agilent technologies, Santa Clara, CA, US). The raw data was normalized using Genespring software (Agilent). Genes with a fold change of at least 1.5 and have statistically significant (*p* < 0.05) were grouped in functional categories based on Gene Ontology database (GO: http://www.geneontology.org/). Functional pathways (Kyoto Encyclopedia of Genes and Genomes, KEGG) were also analyzed in genes with a fold change of at least 2 between two groups.

#### Real-Time Quantitative PCR Analysis

To validate the gene expression data obtained through the microarray analysis, a real-time quantitative PCR analysis was performed. Total RNA from the same samples were acquired. First strand cDNA was synthesized using cDNA Reverse-Transcription Kit (Revert Aid First Strand cDNA Synthesis Kit, K1622, Thermo). Quantitative PCR was performed with GoTaq qPCR Master Mix (Promega, A6001, USA). The expression levels of the target sequences were normalized to those of an endogenous control, glyceraldehyde-3-phosphate dehydrogenase (GAPDH).

### Statistical Analysis

All continuous variables are presented as mean ± standard deviation, and categorical data are expressed as frequencies and percentages. The data were tested for a normal distribution using the Shapiro-Wilk normality test. In cases where the data fulfilled normality test, Student's *t*-test was used to assess the significant differences, and in cases where the data failed the normality test, the non-parametric Mann-Whitney test was used. A two–tailed *P* ≤ 0.05 was considered to indicate statistical significance. ANOVA analysis was conducted for three group comparisons in Echo and others whenever necessary. Statistical significance was indicated by *p* < 0.05. The microarray data was imported into online an SAS analysis system for gene expression data analysis. All statistical analyses were performed using IBM SPSS Statistics 22.0 (SPSS, IBM, USA) and GraphPad Prism software (version 6.0; GraphPad Software, LaJolla, CA).

## Results

### Effect of LLVNS on the Heart Rate

The HR values at baseline, before the VNS device implantation, at 1 h after the VNS device implantation and before sacrifice are shown in Table 1. The HRs were compared between baseline and before sacrifice, and between before and 1 h after the implantation of the VNS stimulators. LLVNS did not show any significant effects on the HR at 1 h after the VNS device implantation and before sacrifice.

**Table 1:** The effect of low level vagus nerve stimulation on heart rate.

**HR (beats min^**−1**^)**	**Sham operation (*n* = 5)**	**MI (*n* = 6)**	**MI+LLVNS (*n* = 5)**
Baseline	128 ± 9	131 ± 14	130 ± 17
Before sacrificed	129 ± 15	133 ± 16	127 ± 15
Before LLVNS device implanted	130 ± 13	140 ± 18	141 ± 20
1 h after LLVNS device implanted	132 ± 13	142 ± 12	138 ± 13

*LLVNS, low level left vagus nerve stimulation; MI, myocardial infarction; HR, heart rate*.

### LLVNS Reduced the Occurrence of Ventricular Arrhythmias

**Figure 2:**
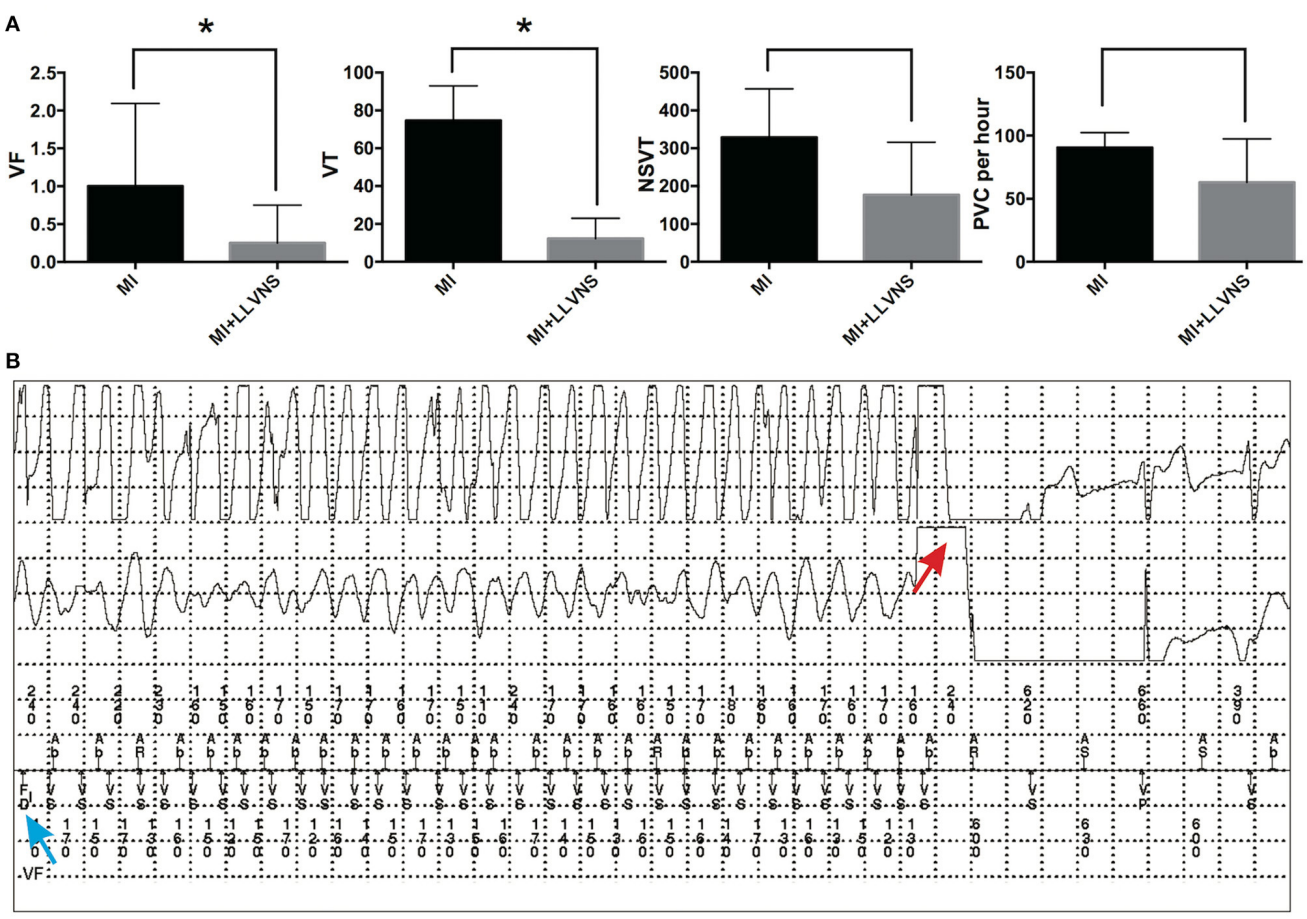
The occurrence of VAs in the MI and MI+LLVNS groups. **(A)** The occurrence of VF, VT, NSVT, and PVC between the MI group (*n* = 6) and the MI+LLVNS group (*n* = 5) (*p < 0.05). **(B)** Example of an IEGM of ventricular fibrillation detected by the ICD and terminated by an ICD shock. The blue arrow shows the VF detected; the red arrow indicates the shock by the device, which restores the sinus rhythm. VAs, ventricular arrhythmias; VF, ventricular fibrillation; VT, ventricular tachycardia; NSVT, non-sustained ventricular tachycardia; PVC, premature ventricular contraction; ICD, implantable cardioverter defibrillators; LLVNS, low level left vagus nerve stimulation; MI, myocardial infarction.

As shown in Figure 2, the episodes continuously recorded via ICDs revealed that compared with the MI group, the MI+LLVNS group exhibited significantly fewer VF (0.25 ± 0.21 vs. 1 ± 0.44, *p* = 0.045) and VT (12.3 ± 5.4 vs. 74.7 ± 7.5, *p* < 0.001) episodes, and the number of NSVT (176.8 ± 70.1 vs.329.2 ± 52.1, *p* = 0.11) and PVC (62.9 ± 17.3 per hour vs. 90.6 ± 4.8 per hour, *p* = 0.18) episodes appeared to be lower but without reaching statistical significance. An example of an intracardiac electrocardiogram of VF detected by the ICD and terminated by an ICD shock is shown in Figure 2B.

### Effect of LLVNS on the Left Ventricular Function

The echocardiography results in the three groups are presented in Table 2. The left ventricular function in both the VNS+MI and MI groups was significantly deteriorated when compared with that in the sham operation group. After 3 weeks of LLVNS, the LVEF in the MI+LLVNS group was significantly higher (44.5 ± 0.4% vs. 26.3 ± 7.5%, *p* = 0.006), and the LVEDD was significantly lower (3.4 ± 0.2 cm vs. 4.2 ± 0.9 cm, *p* = 0.001) than those in the MI group.

**Table 2:** Echocardiographic measurements between the 3 groups.

**Measurement**	**Time**	**Sham operation (*n* = 5)**	**MI (*n* = 6)**	**MI+LLVNS (*n* = 5)**
**LVEF (%)**	Baseline	61.5 ± 2.4	59.7 ± 4.1	60.4 ± 2.4
	3 weeks after MI	61.5 ± 3.2	26.3 ± 7.5^#^	44.5 ± 0.4^*^^#^
**LVEDD (cm)**	Baseline	3.3 ± 0.3	3.2 ± 0.9	3.1 ± 0.4
	3 weeks after MI	3.1 ± 0.5	4.2 ± 0.9^#^	3.4 ± 0.2^*^
**LVESD (cm)**	Baseline	2.1 ± 0.5	2.8 ± 1.0	2.2 ± 0.3
	3 weeks after MI	1.99 ± 0.36	2.8 ± 0.6^#^	2.5 ± 0.3^#^

# *compared with those in the sham operation, p < 0.05*;

* *compared with those in MI group p < 0.05. LVEF, left ventricular ejection fraction; LVEDD, left ventricular end-diastolic dimension; LVESD, left ventricular end-systolic dimension; LLVNS, low level left vagus nerve stimulation; MI, myocardial infarction*.

### LLVNS Suppressed Cardiac Neural Remodeling

**Figure 3:**
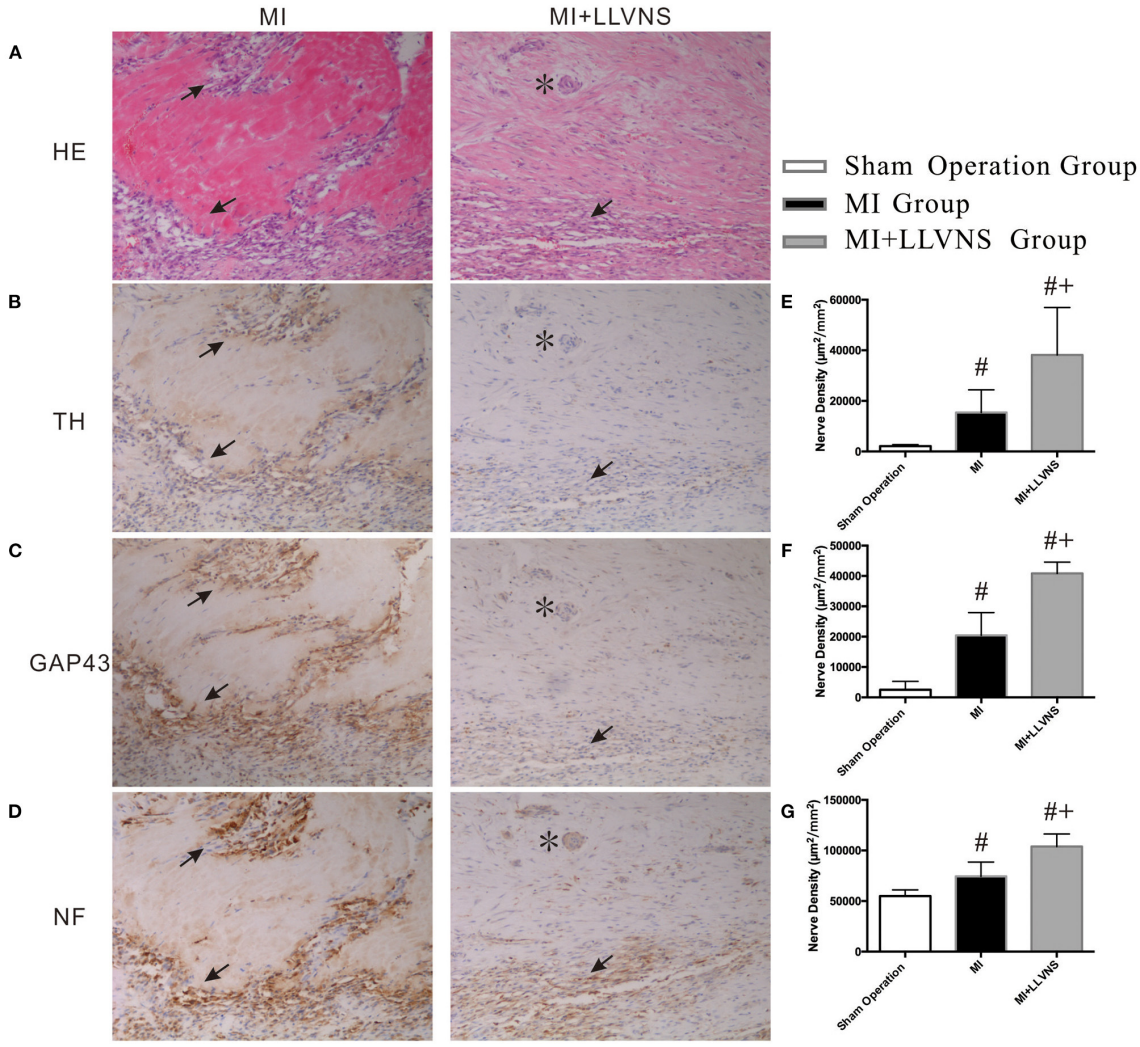
Typical examples and densities of of TH-, GAP43- and NF-positive nerve fibers. **(A)** Border of myocardial injury was stained with hematoxylin and eosin (HE) as revealed by infiltration of inflammatory cells. **(B–D)**: Increased density of TH-, GAP 43- and NF-positive nerve fibers at the border of myocardial injury, respectively. **(E–G)**: Densities of TH-, GAP43- and NF-positive nerve fibers in three groups. The asterisk (*) indicates an artery in the cardiac tissue. Arrows in **(A)** indicate infiltration of inflammatory cells. Arrows in **(B–D)** indicate of TH-, GAP 43- and NF-positive nerve fibers separately. ^#^compared with those in the sham operation, *p* < 0.05. ^+^compared with those in MI group *p* < 0.05. TH, tyrosine hydroxylase; GAP43, growth associated protein 43; NF, neurofilament; LLVNS, low level left vagus nerve stimulation; MI, myocardial infarction. MI group (*n* = 6), MI+LLVNS group (*n* = 5), Sham operation group (*n* = 5).

Typical examples of TH-, GAP43- and NF staining on the border of the myocardial injury in the MI+LLVNS and MI groups are shown in Figure 3. Nerve sprouting was observed in both groups; however, compared with the MI+LLVNS group, the density of the TH, GAP43 and NF positive nerve fibers was higher in the MI group. The densities of the nerve fibers in the infarct area between the three groups are illustrated in Figure 3. Compared with the sham operation group, TH, GAP43 and NF positive nerves were increased in the MI+LLVNS and MI groups 3 weeks after MI. Compared with the MI group, the density of the TH positive (15,411 ± 8,955 vs. 38,180 ± 18725 μm^2^/mm^2^, *p* = 0.023), GAP43 positive (20,436 ± 7,497 vs. 40,870 ± 3701 μm^2^/mm^2^, *p* < 0.001) and NF positive (74,473 ± 14,141 vs. 103,966 ± 12313 μm^2^/mm^2^, *p* = 0.003) nerves in the MI+LLVNS group was remarkably reduced.

### Gene Expression Profiles and Real-Time PCR Confirmation

**Figure 4:**
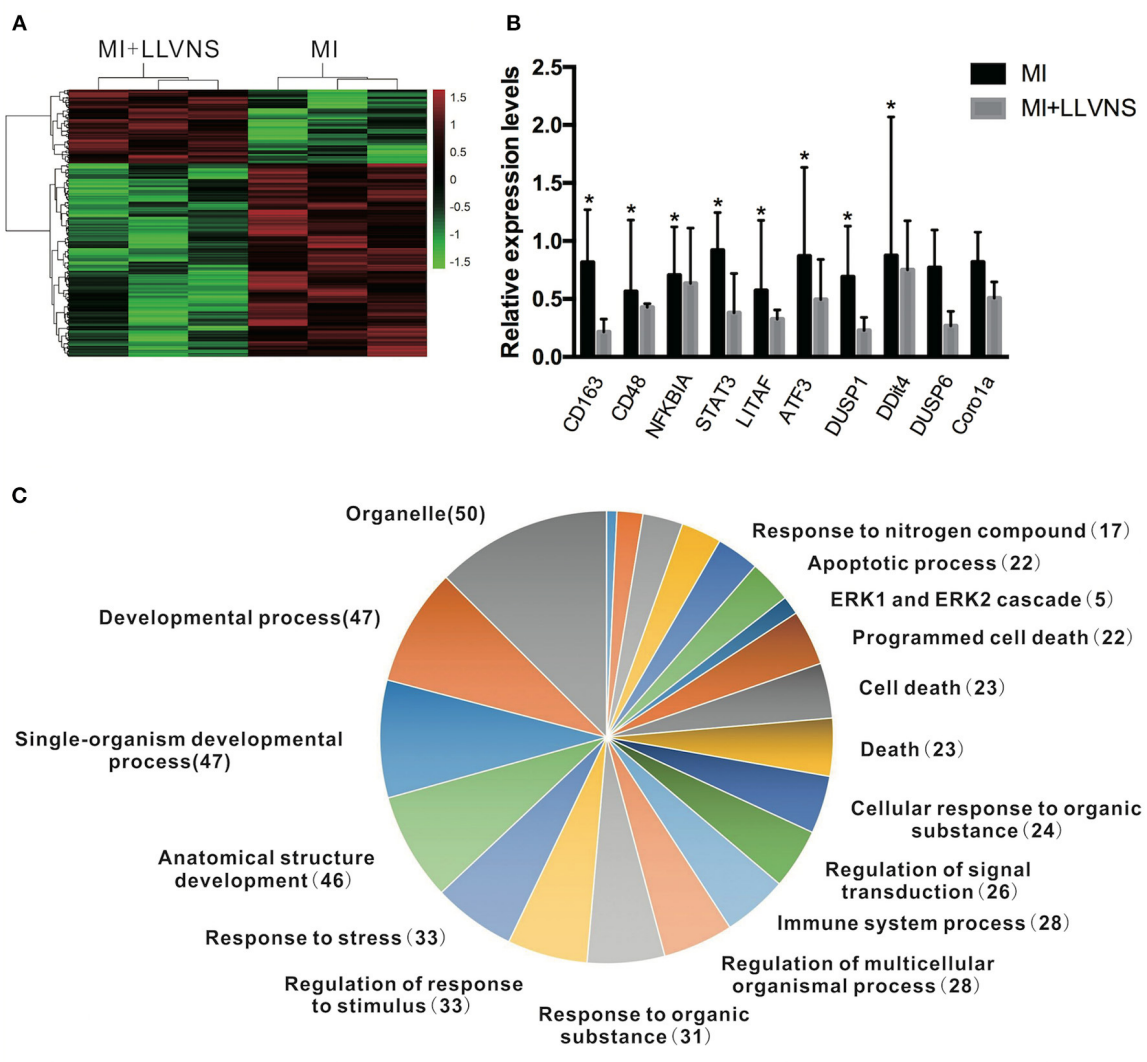
LLVNS regulates the expression of genes. **(A)** The heat map of DEGs in the cardiac tissue (*p* < 0.05, 1.5 fold or more) between the MI and MI+LLVNS groups. **(B)** The verification of some DEGs identified in microarray analyses by RT-PCR analyses. **(C)** A pie chart of gene ontology clustering for the DEGs shown in **(A)**. *p < 0.05. DEGs, differentially expressed genes; LLVNS, low level left vagus nerve stimulation; MI, myocardial infarction. MI group (*n* = 6), MI+LLVNS group (*n* = 5).

**Figure 5:**
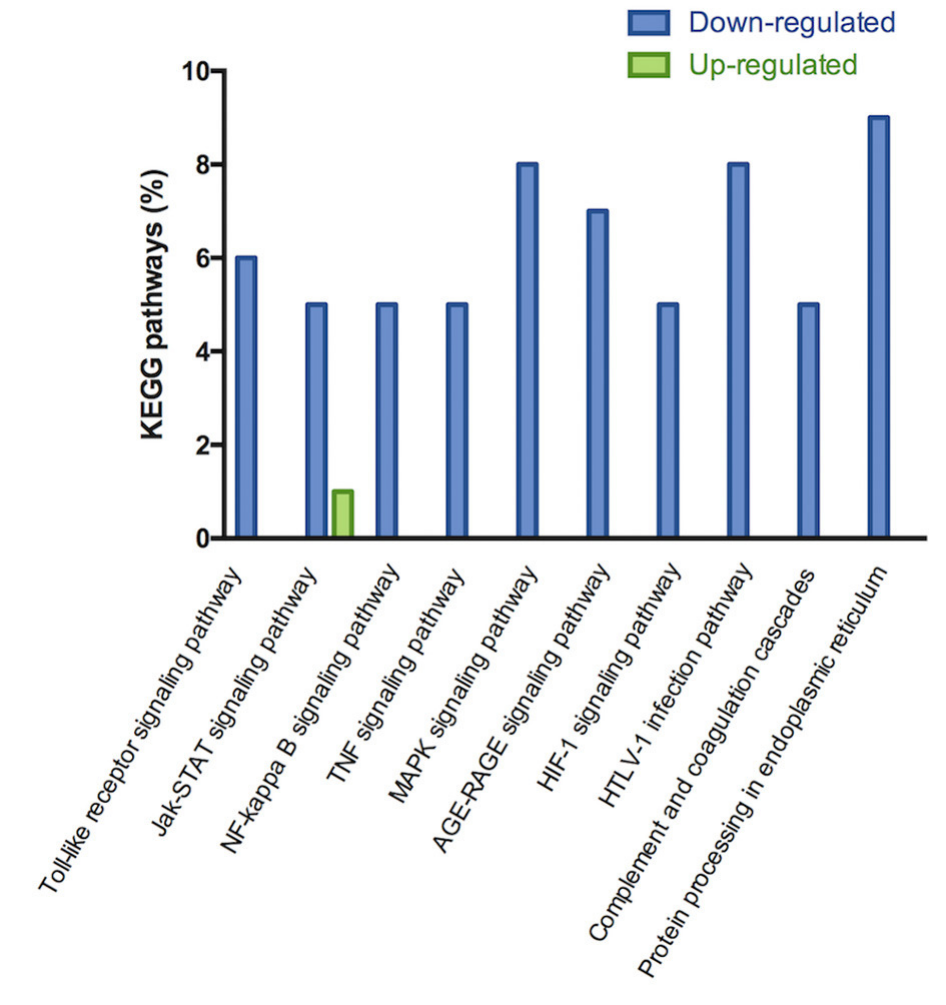
KEGG pathway analysis of up and down regulated genes with a fold change of at least 2 in the dogs of MI+LLVNS group (*n* = 5) compared with MI group (*n* = 6). KEGG, Kyoto encyclopedia of genes and genomes; LLVNS, low level left vagus nerve stimulation; MI, myocardial infarction.

The microarray analysis identified 206 genes out of 20,000 non-redundant predicted genes that were significantly altered by at least 1.5 folds between the two groups (*p* < 0.05) (Figure 4A). Among them, the levels of 151 genes were decreased, and the levels of 55 genes were increased in the MI+LLVNS group, compared with MI group. The results are shown in Supplemental Table 1. The differentially expressed genes (DEGs) were grouped into functional categories based on the Gene Ontology database (GO: http://www.geneontology.org/). The results showed that the majority of DEGs were involved in the regulation of immune system processes, apoptosis processes, programmed cell death processes, the ERK1 and ERK2 cascades, responses to nitrogen compounds, organonitrogen compounds, stress and developmental processes (Figure 4C). Ten expression DEGs identified by the microarray were re-confirmed using quantitative real-time PCR. The relative expression of all the examined genes determined by microarray and real-time PCR were comparable (Figure 4B). Genes with a fold change of at least 2 were defined as functional pathways (KEGG). Based on KEGG pathways, distinct functional classes identified for transcripts were expressed differentially among dogs between the MI and MI+LLVNS groups. Figure 5 shows the relative distribution of 10 KEGG pathways, expressed as the percentage of up- and down-regulated transcripts, between the MI and MI+LLVNS groups. The LLVNS down-regulated transcripts involved in Toll-like receptor signaling, JAK-STAT, NF-κB, TNF, MAPK, and AGE-RAGE pathway.

## Discussion

The main findings of the present canine experimental study are as follows: (1) LLVNS applied at 2 h after MI significantly reduced VAs with improved left ventricular function but without HR changes; (2) LLVNS caused 206 cardiac DEGs, and the effects of LLVNS were likely associated with cardiac neuronal sprouting suppression and decreased inflammation reaction, which could also be explained by the patterns of gene expressions.

In the present study, LLVNS significantly prevented the incidence of VAs, which was consistent with published findings of VNS during myocardial ischemia in animal models (6–9, 21). Lately, Nearing et al. found VNS provided cardiac electrical stability and significantly prevented non-sustained VT in the ANTHEM-HF study (14). In contrast to previous studies, the dose of LLVNS without HR changes in this study could reach the therapeutic target zone reported by previous studies and contributed to satisfactory autonomic engagement for cardiac control (18, 22). The study reported by Shen et al. (23) demonstrated a reduction in atrial fibrillation by LLVNS, while the present study showed a reduction in post MI VAs. Since HR alterations can be avoided using LLVNS, the intracardiac electrode of the stimulator, which was designed to sense the HR in the previous implantable device, (24) may not be necessary. LLVNS simplified the method to achieve better VAs prevention.

Post-MI LV remodeling has already been identified as an important substrate for triggering VAs (25). In the present study, LLVNS improved cardiac function with the evidence of improved LVEF and reduced LVEDD, which was consistent with the outcomes of previous studies (8, 9, 24). VNS has been applied as a therapy for patients with heart failure (24). However, large clinical studies on VNS have had mixed results (10–16). A *post-hoc* analysis showed that various neural targets, VNS doses and HR responses may explain the reasons for different results (17). The optimal dose of VNS is critical in achieving efficacy. Ardell et al. (18) described an approach to determine the optimal dose, yielding physiological augment of vagus nerve activity. Different from previous clinical trials, we used optimal LLVNS dose. Additionally, we explored the onset of HF instead of refractory HF, because nerve and tissue remodeling is always irreversible in refractory HF.

The underlying effect of LLVNS is likely associated with cardiac neuronal sprouting suppression, subduing pro-inflammatory responses and cardiac remodeling during MI.

MI can result in nerve injury (26). Cardiac neural remodeling is one of the important underlying mechanisms of incident VAs after MI. Cao et al. (27) previously confirmed that the increase in sympathetic neural activity after MI and the density of these sympathetic nerves are directly correlated with the incident life-threatening VAs. VNS has been widely studied used for preventing VAs. However, no previous study has observed the effect of LLVNS on post-MI cardiac neural remodeling and related underlying mechanisms. GAP43 is a protein that is expressed when the nerve terminal develops. NF is expressed by axonal and dendritic processes (27). TH is an enzyme that catalyzes the conversion from L-tyrosine to L-DOPA and is a rate-determining enzyme for catecholamine synthesis; thus, TH is used as a marker of sympathetic nerves. In the present study, LLVNS could significantly reduce the density of TH, GAP43 and NF positive nerves in the peripheries of myocardial scars as well as perivascular regions.

While some studies have investigated the protective effects of VNS, regulatory pathways at molecular and gene levels have not been explored. Gene array technologies provide an opportunity to elucidate complex pathophysiologic mechanisms at the gene-expression level. In the present study, the different patterns of gene expressions in the remote zone tissues of the LV free wall in response to LLVNS were found, e.g., 206 genes were considered to be DEGs, and 155 out of which were down-regulated and 51 were up-regulated during LLVNS, compared with the MI group without LLVNS. Many of these DEGs were involved in biological processes, which played important roles in the development and progression of diseases, such as apoptotic process, immune system process, programmed cell death, response to nitrogen compound, stress and negative regulation of ERK1 and ERK2 cascade. These findings may indicate new areas for investigating the potential mechanisms related to VNS. Some of the DEGs were involved in cardiac tissue remodeling, neural remodeling and inflammatory reaction, such as NF-κBia, LITAF, TNFR1A and STAT3 (28–30). However, lots of DEGs were not previously reported in the investigation of VNS, such as CD163, CD48, DDit4, and ADAMTS1, which have been demonstrated close association with ischemic heart disease (31, 32). The present study investigated the possible underlying mechanisms by determining gene expressions at transcription level with an advantage over previous studies. Thus, more ivestigation is warranted to evaluate the consequences of the observed altered gene expressions during MI and LLVNS in the present study (33).

## Limitations

There are four potential limitations we have to admit. First, we didn't measure the the infarct size; second, only remote zone tissues of the LV free wall were analyzed with the method of microarray analysis, but peripheral myocardial scar samples were not. Further studies should be conducted to analyze whether more DEGs exist between the LLVNS+MI and MI groups using peripheral myocardial scar tissue samples; third, the MI mechanism distinctly differs from that responsible for MI among patients in clinical practice. Thus, further research in subjects under different conditions after MI are demanded to confirm the results of this study; fourth, a similar analysis using a larger sample size should be conducted to verify the results of this study.

## Conclusion

The present study demonstrates that LLVNS applied for 2 h after MI significantly reduces the incidence of VAs without HR changes and improves the left ventricular function over a 3-week follow-up period. The potential mechanism is closely associated with suppressing cardiac neuronal sprouting, in addition to the differentially expressed genes involved in cardiac tissue remodeling, cardiac neuronal sprouting and inflammatory reaction. LLVNS delivered without altering HR in early stage of MI may be a novel therapeutic strategy for preventing VAs and protecting cardiac function after MI.

## Data Availability Statement

The raw data supporting the conclusions of this article will be made available by the authors, without undue reservation.

## Ethics Statement

The animal study was reviewed and approved by the Animal Care and Use Committee of the Chinese Academy of Medical Sciences, Peking Union Medical College, Fuwai Hospital and Cardiovascular Institute, Beijing, China.

## Author Contributions

SZhao and SZhang participated in the study design. SZhao, YD, and ZL conducted the animal experiment. XN, MT, and YZ contributed to the analysis and interpretation of data for the work. SZhao drafted the manuscript. YD, XN, MT, YZ, and SZhang critically revised the manuscript. All gave final approval and agreed to be accountable for all aspects of work ensuring integrity and accuracy. All authors contributed to the article and approved the submitted version.

## Conflict of Interest

The authors declare that the research was conducted in the absence of any commercial or financial relationships that could be construed as a potential conflict of interest.
